# Swallowed a Tooth

**Published:** 1893-01

**Authors:** 


SWALLOWED A TOOTH.—A domestic while taking a drink of water swallowed a false tooth, which had been held in position by a metal plate. All efforts to extract the tooth failed, and the woman died of hemorrhage a few days later. At the autopsy it was found that the plate had caused a perforation of the esophagus just above the entrance to the stomach.

